# The Careless Examination of Urine1Read before the annual meeting of the Alumni Association of the University of Buffalo, May 2, 1893.

**Published:** 1893-07

**Authors:** Frederick H. Stanbro

**Affiliations:** Springville, N. Y.


### THE CARELESS EXAMINATION OF URINE.[1]

By FREDERICK H. STANBRO, M. D., Springville, N. Y.

An accurate understanding of urinary analysis in its relation to clinical medicine is absolutely essential for a clear comprehension of any disease. This statement is especially true in typhoid fever, pneumonia, scarlatina, and, in fact, in all the acute blood diseases, and also with acute surgical conditions. In all these instances a careful study of the urine frequently proves to be a more accurate index of the rise and fall of the diseased process than any other single symptom, enabling the physician to foretell with accuracy an approaching improvement, or a threatened exacerbation, which is to speedily terminate in a fatal issue. It places him in a position to know what to expect, and better prepare him for coming emergencies.

The general practitioner believes thoroughly in the examination of urine in certain cases, and analyzes specimens, but he does not do so as a routine practice in each case, nor does he do it as carefully as he might, or should. In a case which, in his judgment, needs any analysis, he asks for a specimen of urine ; an ounce or two is brought to his office in any kind of a bottle, which is set away for an hour of leisure, which may be today and may be tomorrow. Then it is examined for its specific gravity, albumen and sugar, if his bottle of Fehling's solution is not empty. Thus he has made an examination of urine ; and, if the results are negative, no further attention is given to this part of the human economy, a part—the eliminatory part—which is just as important in itself as the feeding or nursing. He does not know how much is being passed daily ; no idea of the amount of solids excreted. After such an examination, do you wonder that the busy practitioner sees no great importance in routine urinary analysis ? He thinks he has learned nothing, has lost his time, and feels that the work was unnecessary, and, after a few such trials, it must be a case of unusual gravity or peculiar symptoms that leads him to make the next examination of urine.

The general practitioner, who is often a country practitioner, in his varied duties and miscellaneous practice, feels himself to be well equipped in all the branches of medicine coming to his daily notice, but exact and thorough in no particular line. He can diagnosticate a pneumonia, but cares little whether it is the upper or lower lobe that is involved. He can diagnosticate a fracture of

---

1. Read before the annual meeting of the Alumni Association of the University of Buffalo, May 2, 1893.

the neck of the femur, but is not particular whether it is intra- or extra-capsular. He can treat successfully a case of typhoid fever, not being perplexed over the question as to whether it is or is not of germ origin. Exactness he leaves to the specialist, and especially exactness in the analysis of urine.

If the patient's urine shows no albumen or glucose, his responsibility is ended; but if albumen or glucose does appear, he does not feel competent, or have the time or apparatus to make a quantitive examination.

This is a mistake which should be overcome. It does not take as much time as he thinks to examine carefully a specimen of urine; less than fifteen minutes will give all the necessary knowledge of each specimen, and any extra time he gives to an obscure case, is not time lost to the patient or practitioner—rather the contrary.

The apparatus needed is cheap and simple of manipulation, and the skill, only that which he uses daily and thinks nothing of.

In this short paper I shall lay stress only on the examination of urine for albumen, glucose, and urea; not that these are the only important substances, but rather the most important, for which a test should be made.

No examination of urine is of any value if the daily quantity is unknown; no finding of albumen or sugar is of value, if the knowledge ends there, and the quantity of albumen or sugar and their daily increase or decrease is not learned. To insure himself from error, the testing must be made not once, but many times, to learn if the abnormal product be permanent or transitory. He might as well examine his patient's lungs once, in a case of pneumonia, and never do it again, or only examine a fractured limb when applying the primary dressing, as to leave the urine after giving it one cursory examination.

Albumen can be easily discovered when present by the much used heat and nitric acid test; but we must take the precaution of filtering the urine, if it is at all cloudy, lest we miss a trace of albumen which may be of extreme importance. Another common source of error is the presence of pus, which, upon testing, shows presence of albumen and leads to wrong conclusions. Urine suspected of containing pus should first be boiled with a few drops of liquor potassæ and filtered before testing for albumen.

For the quantitive examination, the method used by Professor Austin Flint is simple, accurate, and easily made. After testing by heat and $HNO_3$, he pours the tested urine into a graduated cylinder and lets the precipitate settle, after which he estimates the quantity from the graduations as being one-tenth, one-fifth, or one-half by volume. The information gained is sufficient, and the method within the power of all.

Fehling's solution, freshly prepared, or Trommer's test, with cupric sulphate and liquor potassæ, are fairly accurate tests for the presence of sugar. Doubtless, Trommer's test is the safest to use, because cases of diabetes are of common occurrence, and Fehling's solution spoils after standing, and must be freshly prepared for each test.

Dr. Geo. H. Blackham, of Dunkirk, has prepared a formula in which C. P. glycerine is used, which remains perfectly staple and is always ready for use.

W. H. Porter, of the Post-Graduate School, New York, says that for the general practitioner, a fair idea of the increase or decrease of the amount of glucose may be obtained by each day getting the daily amount of urine passed and taking the specific gravity. A more accurate and scientific method is by the yeast test, now made so simple and convenient by using the apparatus of Max Einhorn, of New York. The apparatus consists of a curved tube, one arm of which is enlarged into a bulb, the other arm being graduated. A small amount of compressed yeast is thoroughly mixed in a test tube with the urine, and then poured into the cylinder and set away for twenty-four hours. The following day the $CO_2$ evolved has displaced the urine, and the height of the gas is shown by the markings on the cylinder, which give the per cent. of glucose in the urine. The great advantage of this saccharometer is that you can say the following day whether the urine contains glucose or no; also the quantity of glucose.

Urea is a substance of whose presence too little notice is taken. Its importance cannot be overestimated, as it is the principal product of retrograde changes of nitrogenous materials, and, next to water, is the most abundant and important ingredient found in normal urine. If it be retained in the system, it is a true poison, and if the retention be marked and not relieved, death follows. Measuring the quantity in the urine is a guide to its accumulation in the system and a warning of convulsions or death, as well as a guide to the proper treatment to prevent these catastrophies.

Of the many methods of estimating the amount of urea in urine, the following by Dr. Chas. Doremus, of New York, is the most practical : A curved tube, similar to the saccharometer of Einhorn, is filled to the bulb with a known solution of hypobromite, and then with a pipette the urine is slowly introduced. Rapidly the urea is decomposed and the nitrogen (N) fills the upper end of the tube, displacing the urine into the bulb. When the action has ceased, the graduations show the number of grams or grains of urea to the c. c. or oz., then, by knowing the amount of urine passed during the twenty-four hours, the daily amount of urea is easily found.

Before closing, a word should be said about examination of urinary sediments.

Now-a-days the aid of the microscope in diagnosis is almost a necessity, especially for the general practitioner, who is often at a distance from cities and the help of specialists. In all diseases of the kidneys, the microscope gives much information that cannot be otherwise obtained, and without which the physician is at bay. By the aid of the microscope he gains knowledge by which he can diagnosticate earlier, treat more scientifically, and foretell more accurately than by any other single help.

How better can we judge of the workings of the human engine than, knowing the amount and kind of fuel put in, we learn the exact amount and peculiar kind of waste thrown out? With this knowledge we can accurately judge the action of the body and see for certain whether there is a gain or loss. By an accurate knowledge of the excreta we can know wherein the economy is at fault and what function is not being performed. With such additional knowledge, who can say we are not better prepared to do battle with disease and death ?

How often do obscure diseases grow plain and clear after an examination of the urine, showing, it may be, the cause of the constant vomiting to be an undiscovered and unsuspected Bright's disease, the cause of an attack of so-called asthma to be uremia, or back of a case of gangrene, a system weakened by diabetes ?

Who can doubt or question that benefit comes from systematic examinations of urine in our cases ? How much time would be gained, and, possibly, life saved, if we always examined the urine of our pneumonia patients, our typhoid patients, and especially the urine of the pregnant woman, whose life depends on the care and attention of her physician ?

